# Distinct Antigen Delivery Systems Induce Dendritic Cells’ Divergent Transcriptional Response: New Insights from a Comparative and Reproducible Computational Analysis

**DOI:** 10.3390/ijms18030494

**Published:** 2017-02-24

**Authors:** Valerio Costa, Dario Righelli, Francesco Russo, Piergiuseppe De Berardinis, Claudia Angelini, Luciana D’Apice

**Affiliations:** 1 Institute of Genetics and Biophysics “Adriano Buzzati-Traverso”, CNR, Via P. Castellino 111, 80131 Naples, Italy; valerio.costa@igb.cnr.it; 2 Dipartimento di Scienze Aziendali—Management & Innovation Systems/DISA-MIS, University of Salerno, Via Giovanni Paolo II 132, 84084 Fisciano (SA), Italy; d.righelli@na.iac.cnr.it; 3 Istituto per le Applicazioni del Calcolo, CNR, Via P. Castellino 111, 80131 Naples, Italy; francesco.russo.cib@gmail.com (F.R.); c.angelini@iac.cnr.it (C.A.); 4 Institute of Protein Biochemistry, Consiglio Nazionale delle Ricerche, Via P. Castellino 111, 80131 Naples, Italy; p.deberardinis@ibp.cnr.it

**Keywords:** system vaccinology, RNA-Sequencing, dendritic cells, reproducible research

## Abstract

Vaccination is the most successful and cost-effective method to prevent infectious diseases. However, many vaccine antigens have poor in vivo immunogenic potential and need adjuvants to enhance immune response. The application of systems biology to immunity and vaccinology has yielded crucial insights about how vaccines and adjuvants work. We have previously characterized two safe and powerful delivery systems derived from non-pathogenic prokaryotic organisms: E2 and fd filamentous bacteriophage systems. They elicit an in vivo immune response inducing CD8+ T-cell responses, even in absence of adjuvants or stimuli for dendritic cells’ maturation. Nonetheless, a systematic and comparative analysis of the complex gene expression network underlying such activation is missing. Therefore, we compared the transcriptomes of ex vivo isolated bone marrow-derived dendritic cells exposed to these antigen delivery systems. Significant differences emerged, especially for genes involved in innate immunity, co-stimulation, and cytokine production. Results indicate that E2 drives polarization toward the Th2 phenotype, mainly mediated by *Irf4*, *Ccl17*, and *Ccr4* over-expression. Conversely, fd-scαDEC-205 triggers Th1 T cells’ polarization through the induction of *Il12b*, *Il12rb*, *Il6*, and other molecules involved in its signal transduction. The data analysis was performed using RNASeqGUI, hence, addressing the increasing need of transparency and reproducibility of computational analysis.

## 1. Introduction

In the last three decades, the rapid advances in vaccinology enabled us to prevent some of the major diseases that used to kill or incapacitate millions of children. Currently, the most advanced analyses in such settings rely on transcriptomics, using mostly ribonucleic acids (RNAs) isolated from whole blood or leukocytes; these, combined with proteomics and high throughput sequencing of B and T cell repertoires, have provided new powerful tools to study the perturbations of the immune system induced by vaccination and adjuvant administration [1]. More recently, the scientific community has focused its interest on the definition of transcriptional signatures to study immune responses induced by already existing and candidate vaccines [2]. Since adjuvants are added to vaccine formulations in order to enhance the magnitude—and modulate the quality—of the immune response, several studies have also focused on the effect of adjuvants, combined with vaccines, and the possibility to have antigenic carriers endowed with adjuvant activity in new vaccine formulations is appealing [3].

In previous studies, we established, and largely characterized, two innovative delivery systems derived from nonpathogenic prokaryotic organisms able to induce immune response in vivo: the E2 and the fd filamentous bacteriophage systems [4,5,6,7]. The former is based on the use of the E2 protein of the pyruvate dehydrogenase complex of *Bacillus stearothermophilus* organized as a viral capsid structure. Sixty copies of recombinant E2 protein self-assemble to form a pentagonal dodecahedral scaffold with icosahedral symmetry. It results in the formation of a large multimeric particle with a molecular weight >1.5 Mega Daltons and a diameter of approximately 24 nm. This protein scaffold can be modified on the N-terminus by replacing the natural peripheral domains of E2 with foreign peptides and proteins, creating a novel E2 multimeric antigen display system [8]. We have already demonstrated that the E2 system is able to induce, upon systemic administrations, a strong humoral response in a mouse model, also inducing the formation of neutralizing antibodies against a clade of Human Immunodeficiency Virus (HIV) viruses, when used to display the third variable (V3) loop of the gp120 HIV protein [5,9]. Using a mouse model of mucosal vaccination, we also proved the potential application of the E2 scaffold as an antigen delivery system for mucosal immunization [10]. The second antigen delivery system described here is based on a modification of the phage display technology. The N-terminal region of the major pVIII coat protein of bacteriophage fd virions can be modified to display one or more antigenic epitopes, and this system offers the potential for safe and inexpensive vaccines to elicit full-spectrum immune responses [11]. We have already described that the filamentous bacteriophage antigen display system induces both the innate and the adaptive immune response. When engineered to express antigenic epitopes, it elicits T cell help [12] and triggers a cytotoxic T cell-mediated response [13]. We have further improved this delivery system by targeting fd particles to dendritic cells via DEC-205 (fd-scαDEC-205), an endocytic receptor expressed mainly by dendritic cells [7]. The introduction of a single chain anti DEC-205 antibody on the fd envelope allows the bactriophage to be internalized and to deliver antigens to late endolysosomal compartments enhancing efficiency of antigen presentation by dendritic cells through the induction of their activation *via* TLR9 engagement [14]. We recently demonstrated that fd-scαDEC-205 is a powerful delivery system that induces CD8+ T cell responses even when administered in the absence of adjuvants or maturation stimuli for dendritic cells [14].

Herein, we describe a detailed analysis of one of our RNA-Sequence (RNA-Seq) datasets of bone marrow-derived dendritic cells (BMDCs) upon exposure to these antigen delivery systems and the systematic comparison between them. Taking advantage of these validated immunological models, such a comparative analysis revealed a transcriptional signature that specifies a differential—and unique—ability to induce a distinct immune response. Indeed, data highlighted a more robust transcriptional activation of dendritic cells induced by fd-scαDEC-205, with the coordinated induction of clusters of co-regulated genes, including those encoding proteins of the inflammosome. Interestingly, our analysis also revealed a pronounced switch in glucose metabolism and energy production of dendritic cells pulsed with both the antigen delivery systems, which was not identified before. In Table 1 we have summarized the most relevant genes differentially expressed.

Last but not least, all the analyses of transcriptome data have been performed in the spirit of reproducible (computational) research [15,16,17,18]. In the last decades there has been an increasing need for transparency and reproducibility of computational analysis, to cope with potential mistakes, mis-conductions, and inconsistency that might hamper results of published papers. The problem is particularly relevant for the analysis of complex omics studies [19,20] as several studies have suffered from the lack of reproducibility. The American Society of Cell Biology (ASCB) has encouraged a Data Reproducibility Task Force [21] and several journals are involved in promoting publishing standards for reproducible data. To this aim, thanks to the updated version of the RNASeqGUI [18,22], we released all computational analyses in a transparent and fully reproducible way. The advantage of such approaches is twofold: first, all the steps of the analysis can be investigated and performed by independent researchers since the code used is made available as Supplementary file; second, we describe and comment the code in detail and in a user-friendly spirit. Therefore, beyond the interesting molecular findings for system vaccinology, this work represents a useful guide that can be broadly applied to transcriptomics data in different research fields/contexts and is a way to cope with computational reproducibility issues.

## 2. Results

### 2.1. Perturbation of Gene Transcription and Metabolic Pathways upon Exposure of Dendritic Cells to Both Antigen Delivery Systems

**Figure 1:**
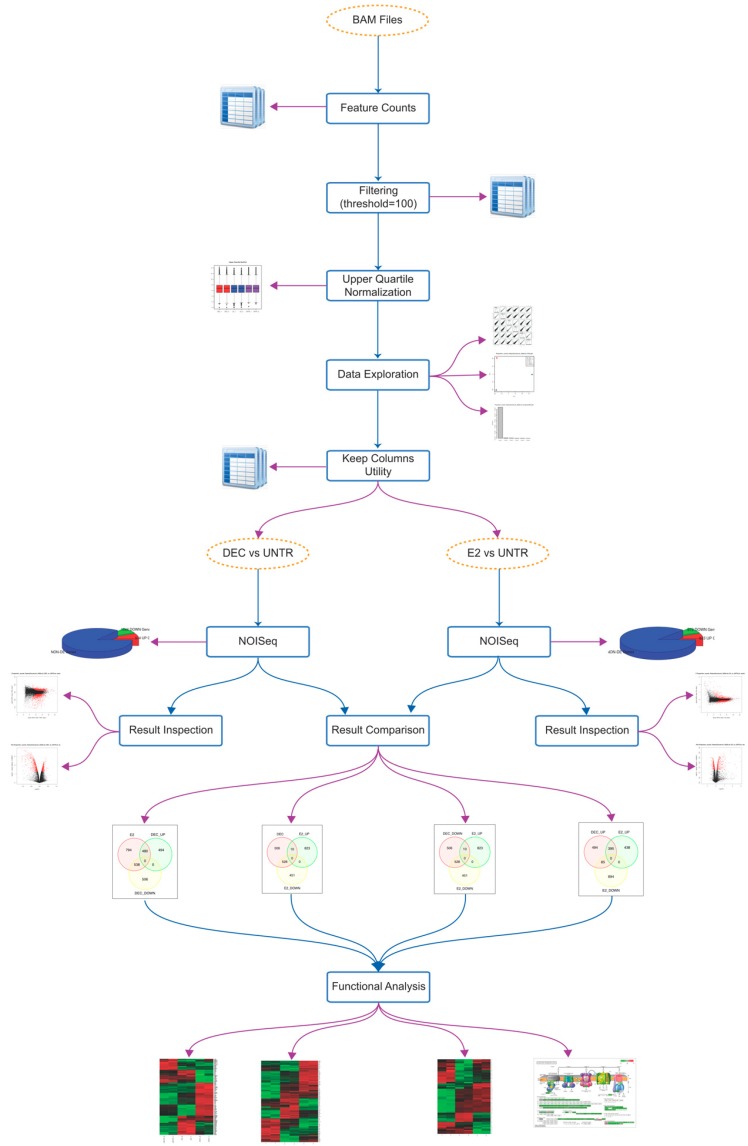
Computational workflow of the RNA-Sequence (RNA-Seq) data analysis. A graphical representation of the entire analysis workflow carried out with RNASeqGUI. Golden ellipses represent most relevant data. Blue boxes represent analysis steps and blue arrows represent the analysis direction. Violet arrows indicate a produced output. BAM: Binary (Sequence) Alignment/Map data format, UNTR: untreated cells, NOISeq: R/Bioconductor package [24].

Bone marrow-derived Dendritic Cells isolated from C57 mice were pulsed with PBS or two distinct antigen delivery systems, E2 and fd-scαDEC-205 (Supplementary Figure S1A) and, 20 h later, changes in transcriptome were detected using RNA-Sequencing (Supplementary Figure S1B). Data were comprehensively and systematically analyzed using the new implementation of the software RNASeqGUI (Figure 1).

**Figure 2:**
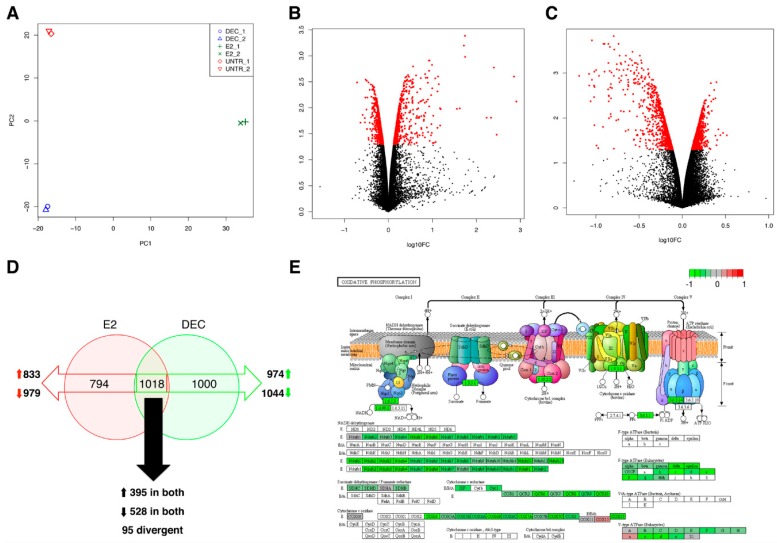
Gene expression analysis of stimulated dendritic cells. (**A**) Principal component analysis of filtered and normalized read counts. Clustering of replicates of bone marrow-derived dendritic cells (BMDCs) treated with PBS (UNTR_1 and UNTR_2), pulsed with E2 (E2_1 and E2_2) and fd-scαDEC-205 (DEC_1 and DEC_2) upon PCA are shown. (**B**,**C**) Volcano plots show, in red dots, the genes that are differentially expressed (red dots, PP > 0.95) in BMDCs stimulated with E2- (panel **B**) and fd-scαDEC-205 (panel **C**) in pairwise comparisons vs. untreated cells. (**D**) Comparison of the differentially expressed genes on BMDCs upon stimulation with E2 (red circle) and fd-scαDEC-205 (green circle) using Venn diagrams. Empty red and green arrows indicate genes with altered expression upon E2 and fd-scαDEC-2055 stimulation, respectively. Black arrows indicate the genes whose expression is affected upon exposure to both antigen delivery systems. (**E**) Graphical representation of the pathway analysis results for genes of the oxidative phosphorylation pathway (OXPHOS in Kyoto Encyclopaedia of Genes and Genomes (KEGG) database) that are modulated upon stimulation with both antigen delivery systems. As an example, genes that are down-modulated upon fd-scαDEC-205 exposure are depicted. Color intensity is proportional to the fold-change.

A high correlation between all technical replicates was observed, whereas a marked difference in global gene expression profiles of DCs pulsed with the two stimuli and untreated DCs was evident by the principal component analysis (PCA; Figure 2A). Independent analyses of differential expression were performed to rule out the effect of exposure from both antigen delivery systems on BMDCs. Volcano plots in Figure 2B,C, generated in the *Result Inspection Interface* of the graphical user interface (GUI), revealed the extent of differential expression per condition. As depicted in Figure 2D, 1812 genes were differentially expressed in BMDCs upon E2 exposure (833 up- and 979 down-regulated; Posterior Probability (PP) > 0.95), whereas 2018 were significantly altered by fd-scαDEC-205 (974 up- and 1044 down-regulated; PP > 0.95).

Interestingly, 395 genes were induced in DCs upon the exposure to both antigen delivery systems (PP > 0.95; Figure 2D). Gene ontology analysis revealed a statistically significant enrichment in “chromosome organization” (33 genes) and “regulation of transcription” (77 genes) ontology terms (*p*-value adjusted (*p_adj_*) < 0.05). In particular, data indicated the induction of genes belonging to the mediator complex (*Med12*, *Med13*, *Med13l* and *Med14*), to the Notch signaling (*Notch1* and *Notch2*), as well as to different genes encoding transcriptional regulators (zinc finger proteins). Moreover, pathway analysis did not reveal any significantly enriched pathways, however, a relevant fraction of induced genes are involved in “focal adhesion” that is generally associated to dendritic cell activation and cell remodeling.

Finally, 528 genes were down-regulated in DCs upon both treatments (PP > 0.95). Pathway analysis, carried out with DAVID and GAGE tools in the GUI, showed a significant enrichment in “Oxidative phosphorylation” (OXPHOS) and “Ribosome” processes (Figure 2E). The down-regulation of OXPHOS-related genes—that resembles the Warburg effect widely described in tumor cells—is in line with the activation of dendritic cells by a stimulus. Indeed, DC activation is known to induce a metabolic switch from OXPHOS to glycolysis, followed by a significant decrease in tricarboxylic acid (TCA) cycle activity [23].

### 2.2. RNA-Seq Identifies Antigen Delivery System-Specific Gene Expression Signatures

**Figure 3:**
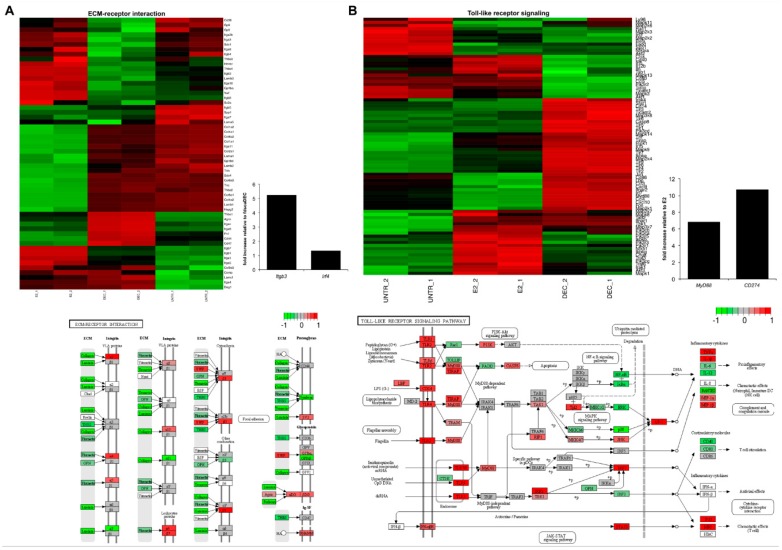
Biological pathways affected upon dendritic cell stimulation with the two antigen delivery systems. Genes differentially expressed upon stimulation with (**A**) E2 and (**B**) fd-scαDEC are depicted. “Extracellular matrix (ECM)-receptor interaction” and “Toll-like receptor signaling” are illustrated as the most perturbed pathways upon E2 and fd-scαDEC exposure, respectively. Heat maps (upper panel) of differentially expressed genes in BMDCs treated with PBS (UNTR_1 and UNTR_2) then pulsed with E2 (E2_1 and E2_2) and fd-scαDEC (DEC_1 and DEC_2) are depicted along with graphical representations of the most significant Kyoto Encyclopedia of Genes and Genomes (KEGG) pathways (lower panel). In the heat maps, the degree of differential expression is indicated by a three-color code (down-regulated genes in green, up-regulated in red, and those with little-to-no variation in black). In each panel, bar graphs report the results of the validation analysis by qReal-Time PCR. Expression data are reported as a relative expression compared to BMDCs stimulated with (**A**) fd-scαDEC-205 and (**B**) E2 system.

As we aimed to identify antigen delivery system-specific gene expression signatures we focused on the analysis of genes that were altered in DCs only upon a given stimulus or upon both treatments but with a different trend of expression (i.e., up-regulated by E2 and down-regulated by fd-scαDEC-205 and vice versa). However, only ten genes were induced upon E2 exposure and down-modulated by fd-scαDEC-205. Conversely, we found a large fraction of genes induced exclusively by the E2 system (428 genes; PP > 0.95). The vast majority of them belong to “Extracellular Matrix-receptor interaction”, “Focal adhesion”, and “Regulation of actin cytoskeleton” pathways. Expression of surface molecules involved in cell migration, such as integrins, was validated by qReal-Time PCR, as shown in Figure 3A for the *Itgb3* gene, confirming RNA-Seq data. Additionally, gene ontology analysis revealed that “Regulation of signal transduction” and “Regulation of cell communication” were two of the most enriched ontology terms. This finding is in line with a general mechanism of cell cytoskeleton remodeling and with the antigen processing and presenting capability of DCs. Thus, our data indicated a pronounced activation of genes related to adhesion and extracellular matrix remodeling and a less robust activation of immune-related genes in DCs upon E2 exposure.

Conversely, pathway analysis and gene ontology of the subset of fd-scαDEC-205-specific genes (494 genes, PP > 0.95) revealed a more robust transcriptional induction, with the activation of several genes associated to DCs’ maturation. The significant enrichment of “Toll-like receptor”, “NOD-like receptor” (Nucleotide-binding Oligomerization Domain-like receptors) “and RIG-I-like receptor” (Retinoic acid-Inducible Gene I-like receptor) signaling pathways (Figure 3B) confirmed our previous observation [14]. Experimental validation of *Myd88* (Myeloid differentiation primary response) gene induction—one of the main players of Toll-like receptor pathways—confirmed the observation (Figure 3B). Interestingly, overall the RNA-Seq data indicated that danger-sensing receptor pathways are not affected by E2 exposure, confirming that the two antigen delivery systems activate DCs through distinct molecular mechanisms.

**Figure 4:**
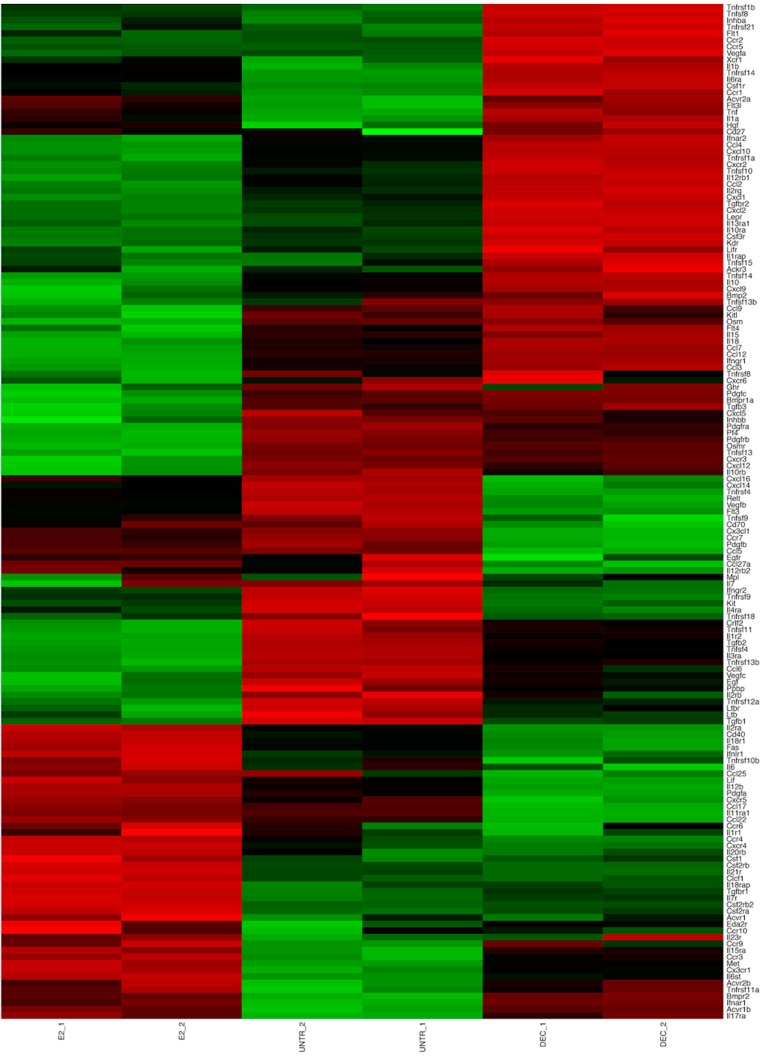
Differential expression of genes belonging to the Cytokine-cytokine receptor interaction pathway in dendritic cells pulsed with the two distinct antigen delivery systems. The heat map shows the differential expression of genes belonging to the “Cytokine-cytokine receptor interaction” pathway in BMDCs treated with PBS (UNTR_1 and UNTR_2), and pulsed with E2 (E2_1 and E2_2) and fd-scαDEC-205 (DEC_1 and DEC_2). The degree of differential expression is indicated by three-color codes (down-regulated genes in green, up-regulated genes in red and those genes with little-to-no variation in black).

Finally, in line with the notion that DCs’ activation and maturation is followed by a prompt activation of a specific transcriptional program, the pathway analysis revealed that both delivery systems trigger cytokine production. Interestingly, we could observe a differential pattern of induction. Indeed, as shown in Figure 4, upon stimulation of DCs with the antigen delivery systems, we observed specific chemokine receptor expression signatures. The transcriptional changes following the stimulus with fd-scαDEC-205 clearly recapitulate the maturation effect induced by this antigen delivery system, as described elsewhere [14,25]. Over-expression of the *Cd274* gene encoding a co-stimulatory DC molecule—confirmed in this work by qRT-PCR—strongly indicated the pronounced DCs’ activation (Figure 3B). Additionally, RNA-Seq data also showed the marked down-modulation of the *Ccr6* gene, encoding a known marker of immature DC, and the simultaneous transcriptional induction of the inflammatory chemokines *Cxcl10*, *Ccl2*, *Ccl3*, *Ccl4*, *Ccl5*, and of the chemokine receptors *Ccr1*, *Ccr2* and *Ccr5*, in line with the strong maturation stimuli described by Foti and colleagues [26]. Remarkably, the above-mentioned genes are down-modulated in DCs upon stimulation with the E2 particles, highlighting that a different transcriptional program follows E2 stimulation. We also report that *Il1a* and *Il1b* gene transcripts are significantly induced in DCs by fd-scαDEC-205 as well as *Il18* and the genes encoding the interferon alpha receptor 1 and 2. In line with the pattern of DCs’ maturation, the *Cxcl12* gene—encoding the chemotactic factor SDF1 (Stroma Derived Factor 1), responsible of DCs’ homing—is significantly induced by fd-scαDEC-205 exposure. Conversely, genes encoding homeostatic chemokines such as *Ccl25* and *Ccl27* and those encoding their membrane receptors, *Cxcr5*, *Ccr9*, and *Ccr10*, are highly induced by E2 particles (Figure 4).

The analysis of gene expression signatures of the chemokine–chemokine receptor pathway strongly indicates that the two different antigen delivery systems induce distinct membrane receptors and secreted molecules, in turn driving a differential polarization of the adaptive immune response. In particular, our results highlight that the E2 system drives polarization toward the Dendritic Cell type 2, since the overexpression of the transcriptional regulator *Irf4* (Figure 3A) of the *Ccl17* gene and of its receptor *Ccr4* have been associated with the Th2 phenotype [27,28]; in contrast, RNA-Seq data clearly show that fd-scαDEC-205, through the induction of *Il12b* (and of its receptor *Il12rb*), *Il6* and the molecules involved in its signal transduction (such as *Il6st*), as well as *Ccl3*/*Mip1a*, is capable of inducing Th1 T cell polarization [29]. Interestingly, the stimulation with fd-scαDEC-205 also induces the transcription of the leptin receptor encoding gene (*lepr*), which is involved in DCs’ survival.

Notably, our analysis also revealed that a fraction of cytokine/chemokine encoding genes are quite similarly modulated by both the antigen delivery systems. Indeed, genes encoding homeostatic chemokines responsible for basal migration such as *Ccl6*, *Cxcl14*, and *Cxcl16*, are down-regulated upon exposure both to E2 and fd-scαDEC-205, though to a different extent. Similarly, *Il7* and *Pdgfra* genes are down-modulated upon both stimuli, probably because these molecules are involved in immune cell survival.

### 2.3. fd-scαDEC-205 Induces the Transcription of a Coordinated Network of Immune-Related Genes

**Figure 5:**
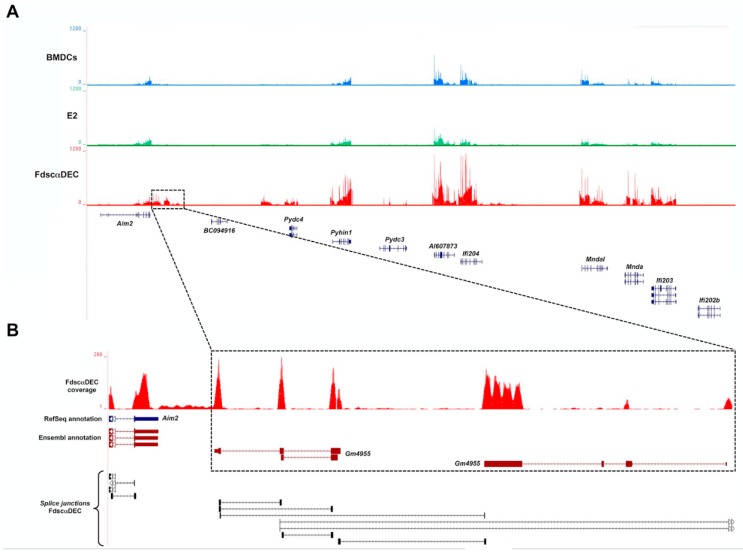
Graphical representation of the cluster of HIN (Hemopoietic Interferon Inducible)-200 family interferon-induced genes on mouse chr 1qH1-H3. (**A**) Screenshot of the University of California Santa Cruz (UCSC) Genome Browser session showing the entire genomic region that encompasses genes encoding proteins of the Interferon inducible p200 family. Coverage of RNA-Seq reads for the BMDCs treated with PBS or pulsed with E2 or fd-scαDEC-205 are indicated by colored vertical bars in blue, green, and red, respectively. Genes annotated in National Center for Biotechnology Information (NCBI) Reference Sequence (RefSeq) database are shown in blue in the lower part. (**B**) Detail of the genomic region showing the expression of the *Gm4955* gene, induced exclusively upon exposure to fd-scαDEC. Genes annotated in Ensembl database are shown in purple.

The systematic comparison of transcriptome data revealed a divergent transcriptional response of DCs upon stimulation with the two distinct antigen delivery systems. Since we observed a more robust and pronounced transcriptional induction of immune-related genes in DCs stimulated by fd-scαDEC-205, we examined whether these genes were over-represented at distinct *loci* in the mouse genome, potentially representing clusters of co-regulated genes. Using the positional enrichment analysis [30], we found that chr1, chr5, and chr19 are particularly enriched for differentially expressed genes (adjusted *p*-values << 0.01). Interestingly, the cluster mapping on 1qH1-H3 is enriched in genes encoding proteins of the HIN-200 family (the Hemopoietic Interferon inducible p200), related to the Interferon response and to the exogenous deoxyribonucleic acid (DNA) detection in the nucleus and cytoplasm (Figure 5A). In mice, immune response mediated by the HIN-200 proteins has been poorly studied even though the interferon-induced (Ifi) proteins have been described as stimulated by IFN type I or II treatment. Here, we report the up-regulation of the entire cluster of *Ifi* genes, as shown by RNA-Seq data in Figure 5. Notably, the same genes are not induced upon E2 exposure. Remarkably, we observed a significant over-expression for *Ifi206* mitochondrial RNA (mRNA), the last member of the HIN-200 family to be identified (Figure 5B). This finding reveals, for the first time to the best of our knowledge, the expression of this gene in professional antigen-presenting cells as well as its transcriptional induction in DCs upon stimulation with an antigen delivery system.

The large cluster of genes mapping to chr5qE3-G1 includes several downstream type I interferon-activated genes with anti-viral activity (*Oas1a*, *Oas1b*, *Oas1c*, *Oas1g*, *Oas2*, *Oas3* and *Oasl1*), early-secreted chemokines with chemotactic effect on neutrophils, cytotoxic T cells, and natural killer cells (*Cxcl1*, *Cxcl2*, *Cxcl3* and *Cxcl10*), and the *Tlr1* gene encoding the Toll-like receptor 1 protein. Additionally, on chr19 we found a cluster of genes including—among others—the one encoding the Cd274 co-stimulatory molecule, two interferon-induced genes (*Ifit1* and *Ifit2*), a guanine nucleotide exchange factor crucial to the regulation of the GTP Hydrolase enzyme (GTPase) CDC42 in mouse DCs (*Dock8*), and a regulator of chemotaxis of Bone Marrow-derived neutrophils and dendritic cells in vitro (*Gnaq*).

## 3. Discussion

The immune response to pathogens involves the coordinate effort of several actors playing different roles. The host-response interaction induces a strong modification of the transcriptional profile of the host immune cells, and the analysis of the host-pathogen interactions available up to now have highlighted the presence of unique and specific transcriptional programs [31].

The development of new deep sequencing technologies has contributed to a collection of a huge volume of data regarding the complex modification in gene expression pattern after host-pathogen interaction, enabling us to define molecular signatures for different pathogens. Mirroring the host-pathogen interactions, different vaccine formulations induce distinct transcriptional profiles depending on the pathogen selected, the adjuvant formulation, the target cell type, and the administration route [32]. Generally, adjuvants are natural ligands or synthetic agonists for pattern-recognition receptors (PRRs). Among them, the Toll-like receptors (TLRs), C-type lectin-like receptors, and the cytosolic NOD-like receptors are able to sense a broad range of microbial stimuli, and the cytosolic RIG-I-like receptors sense viral nucleic acids [25]. PRR activation stimulates the production of pro-inflammatory cytokines/chemokines and type I IFNs that increase the host’s ability to eliminate the pathogen. Thus, the incorporation of pathogen associated molecular patterns (PAMPs) in vaccine formulations can improve and accelerate the induction of vaccine-specific responses.

After vaccination with inactivated viral vaccine, the most prevalent responses are mediated by IFN, while inactivated bacterial vaccines induce a wider spectrum of response such as the induction of the inflammosome response and dendritic cell maturation.

From the several studies published in the field of system vaccinology, it is evident that different pathogens start a common defense response named the host signature, which is shared by different cell types and induced by different pathogen species. It is identified as the “alarm signal” of infection [2]. The IFN-related genes are among the most prominent response genes. Moreover, activation of antigen presentation, lymphocyte maturation, cell adhesion molecule overexpression, and extravasation are the most registered responses. The alarm signal as a transcriptional signature of immune response is mediated by transcriptional regulators and cytoplasmic molecules responsible for the defense mechanism activation.

Since there is urgent need for vaccines against diseases that have not been vanquished yet, and each pathogen raises a different challenge for the immune response, a complete dissection of the response generated by the antigen delivery system can give new hints to vaccine design. Vaccine delivery is the presentation of target antigens to the immune system in order to elicit immune responses appropriate for protection against a specific disease. One of the approaches is based on mimicking natural pathogens as viruses and prompting first the innate response and then the adaptive immune response [33].

In this paper, we describe the systematic comparison of professional transcriptomes of antigen presenting cells after challenging them with two antigen delivery systems, both well characterized for their ability to induce an immune response when administered in vivo in a mouse model [3,5].

Due to the role of scavenger cells and their ability to engulf particles, DCs uptake antigens which they sense as a danger through pattern recognition receptors (PRR). This leads to a dramatic change in cell morphology, metabolism, and induction of cytokine production that we have systematically analyzed at the transcriptional level using RNA-Sequencing.

From the data collected, and from the evidence already published, it is clear that the two systems, once engulfed by dendritic cells, share a common ability to induce modification of the cytoskeleton, changing the cell shape and leading to the lymphoid organ migration. Remarkably, both the systems are capable of inducing a switch in the metabolic pathways of dendritic cells. It has been reported that the metabolic requirements of an activated DC are distinct from those of a quiescent cell and, as such, changes in metabolism must be integral to their successful activation. It is reasonable that DCs use glucose to synthesize fatty acids that are subsequently oxidized, as recently shown to be essential for the development of memory CD8+ T cells [34,35].

In contrast, it is clear from our data that the delivery systems induce a divergent transcriptional program that leads to peculiar downstream effects. Despite the limitation of RNA-Seq studies—i.e., the lack of knowledge about protein levels or protein-protein interactions and modifications (phosphorylation, degradation etc.)—we observed and measured a differential response of stimulated DCs in terms of transcriptional induction of genes encoding cytokine, chemokine, and chemokine receptors. Such a divergent downstream transcriptional program explains the activation of a different immune response. We previously reported that the E2 antigen delivery system is able to induce a strong humoral response [4,5]. Here, we describe this molecule as able to mount a Th2 immune response, while the fd-scαDEC-205 system is a stronger transcriptional inducer in DCs, leading to a Th1 response and to the activation of the Interferon related gene cascade; this latter response is MyD88 dependent and Toll Like Receptor (TLR) 9 mediated [14].

It would be important to dissect the molecular mechanism which drives the E2 or the fd systems to respectively polarize a Th2 versus a Th1 type of response. Many cell subsets involved in the innate and/or adaptive immune response together with the compositions and administration route would likely contribute to Th polarization. We describe here a simplified condition, where the response of a single cell type, the antigen presenting cell, is analyzed, leaving out the complexity of the cross-talk among the several cell types coordinating their role in the immune response; from the data here reported, we suggest a correlation between the change in gene expression and the immune response outcome. Future studies are needed to complete the puzzle in order to explain how immune responses are orchestrated.

Collectively, the model that we propose based on our previous reports [5,10,14,25] and on data emerging from this comparative analysis is: the fd bacteriophage targeted to the dendritic cells via the DEC-205 receptor exactly recapitulates what happens after a viral infection, activating the cytosolic DNA sensors and in turn the inflammosome and the Il-1b production.

It has been reported that DCs activated in vivo in response to type I IFN signaling undergo a metabolic switch followed by increased transcription of the *Hif1a* gene which encodes the hypoxia-inducible factor 1 [35]. Accordingly, RNA-Seq indicated that fd-scαDEC-205, but not E2, is able to significantly increase *Hif1a* expression. Moreover, although not reaching the significance threshold, the transcription of the *Nos2* gene, encoding the inducible nitric oxide synthase (iNOS), was increased by more than 2-fold in DCs after fd-scαDEC-205 exposure (PP > 0.80). Again, E2 failed to stimulate the expression of this gene. The production by activated DCs and macrophages of nitric oxide, a reactive nitrogen species, inhibits mitochondrial respiration and is one the main driving events in the switch from OXPHOS to glycolysis [23]. Taken together, all these observations strengthen the hypothesis that—at least at the molecular level—the activation of DCs by the two antigen delivery systems induces differential pathways and strengthens the assumption that different antigen delivery systems should be selected to induce distinct immune response, depending on the pathogen and on its interaction with the host.

Finally, all our analyses have been carried out in the spirit of reproducible (computational) research to enhance transparency of the findings, allowing other users to re-execute all the analyses, and providing guidelines for similar types of analyses. We want to stress this, since the lack of reproducibility has garnered much attention in the past few years. The analysis of Next Generation Sequencing data, as the RNA-seq considered in this study, involves several steps such as alignment, preprocessing, statistical methods, and pathway analysis. Overall, in the *omic* science era, all the data analyses are increasing in terms of complexity, so it is becoming very difficult to keep track of all steps and parameters used to obtain a finding. The classical textual descriptions in which the “material and method” sections describe the data analysis are no longer adequate to assure data reproducibility and to trace back the precise steps from the raw data to the published results, such as figures and tables. Therefore, we propose to release together with the published papers, dynamic reports describing all the steps carried out and linking results and tables to raw data and code (as done in Supplementary file 1). Such reports can be easily obtained using modern user-friendly tools such as RNASeqGUI, or using literate statistical programming and good computational practices and guidelines [36].

Therefore, as a final aim, we want to promote and encourage the development of novel tools supporting reproducible research and the use of best computational practice guidelines.

## 4. Material and Methods

### 4.1. Antigen Delivery Systems Purification and Cell Culture

The recombinant fd-scαDEC-205 bacteriophage (expressing a single chain variable fragment against mouse DEC-205 molecule) and recombinant E2 protein were purified as previously described [5,7]. Both antigen delivery systems were purified from Lipopolysaccharides contamination by extraction with Triton X-114 (Sigma-Aldrich, Milan, Italy), according to Aida and colleagues [37]. The final particles were tested for endotoxin using the Limulus Amebocyte Lysate (LAL) Assay (QCL-1000, Lonza, Basel, Switzerland), according to the manufacturer’s instructions, and the endotoxin levels were less than 0.05 EU/mL in all preparations.

BMDCs were produced from precursors isolated from the bone marrow of C57BL/6 mice by culturing them with recombinant murine Granulocyte-macrophage colony-stimulating factor (Peprotech, Rocky Hill, NJ, USA) in RPMI (Roswell Park Memorial Institute medium) 1640 (Lonza) medium supplemented with 10% Fetal Calf Serum, 100 Units/mL penicillin, 100 g/mL streptomycin, 1 mM sodium pyruvate, and 55 M 2-mercaptoethanol (GIBCO, Life Technology, Monza, Italy). Cells were collected at day seven of culture, assayed for their phenotypes of dendritic cells, and co-cultured with PBS or the two above-mentioned carriers for 20 h. Animal studies were approved by our institutional review board, and the animal procedures were performed according to rules approved by the ethics committee (permission n. 137/2006-A).

### 4.2. RNA, Library and Sequencing

Isolation of RNA from BMDCs, library preparation, and sequencing have been described previously [10,14]. Briefly, RNA was isolated from cells collected in Tri Reagent (Sigma-Aldrich) according to the manufacturer’s instructions. RNA integrity, quality, and quantity were assessed using Experion (Bio-Rad, Milano, Italy) and NanoDrop1000 spectrophotometer (Thermo Fisher Scientific, Life Technology, Monza, Italy). Illumina TruSeq RNA Sample Preparation Kit (Illumina, San Diego, CA, USA) was used to prepare paired-end libraries that were sequenced at high coverage (100 × 2 bp) on the Illumina HiSeq2000 platform available at the Tigem Institute in Pozzuoli (Naples, Italy).

### 4.3. Data Analysis

RNA-Sequencing datasets used in this manuscript are available in the Gene Expression Omnibus (G.E.O.) database (http://www.ncbi.nlm.nih.gov/geo/) with the identifier GSE60231. In particular, we used the following samples: GSM1468376, GSM1468381, GSM1468388, GSM1468389, GSM1468390, and GSM1468391. However, in this work we have re-analyzed all the samples starting from raw reads. In detail, short paired-end reads (100 × 2 bp) were aligned on the mouse genome mm9, NCBI annotation version 37.67, and on the Ensembl gene annotation (v67) using TopHat version 2.0.14 [38]. Only uniquely mapped reads were retained for further studies. Details can be found in Supplementary File 1. Analysis of aligned sequences was performed using RNASeqGUI [15,19]. RNASeqGUI automatically supports a fully reproducible data analysis. It automatically produces a dynamic report describing all the steps carried out during RNA-Seq data analysis and links results and tables to raw data and code in R language. Each code chunk can be re-executed for the sake of reproducibility. For the current study, we set up a computational pipeline for the analysis of RNA-Seq datasets (Figure 1) and we used RNASeqGUI ver 1.1.2 to perform all the major steps. In brief, we performed gene expression quantification, filtering, and normalization, differential expression analysis, comparison of the results and pathway/gene ontology analysis. To ensure full reproducibility of the entire analysis, all the specific functions, parameters, and additional annotations files are available at http://bioinfo.na.iac.cnr.it/BMDC_analysis/index.html.

Gene quantification was carried out comparing two distinct counting methods implemented in RNAseqGUI (i.e., *SummarizeOverlaps* and *FeatureCounts*). Since very high correlation between the methods was observed (data not shown), *FeatureCounts* option was used in the proposed pipeline, as it is less computationally demanding. Afterwards, genes with a coefficient of variation lower than 100 were filtered out using the filtering procedure in the RNASeqGUI dedicated interface. Then, filtered counts were normalized using the Upper Quartile option in the *Normalization interface* and resulting data have been inspected using Principal Component Analysis and Scatter Plot Matrix functions, available in the *data Exploring interface.* To perform a parallel differential expression analysis for the two distinct delivery systems, the *Keep columns* utility was used to separate normalized count files. Such files were independently processed using the NOISeq algorithm [24], with technical replicates option and *0.95* probability (posterior probability, PP) threshold.

The output lists with differentially expressed genes were compared using three sets of the Venn diagram routine available in the *Result comparison interface*, which automatically saves all intersection gene lists and graphical outputs. Finally, the resulting lists were used for Gene Ontology (GO) term and Pathway analysis with DAVID and GAGE packages implemented in the *Functional annotation* RNASeqGUI section.

DAVID analysis was performed on the KEGG database and on GOTERM_BP_ALL and GOTERM_MF_ALL categories, whereas in the GAGE interface *mmusculus* and *conversion from ENSEMBL ids* have been used, both for pathway and GO analysis. Heat maps were generated using the GAGE dedicated interface section.

### 4.4. Data Validation

Experimental validation of differentially expressed genes was performed by quantitative Real-Time assays. Total RNA was isolated as described in paragraph 2.2. For each sample, 500 ng of total RNA were reverse-transcribed into complementary DNA (cDNA) using the High-Capacity cDNA Reverse Transcription Kit (Life Technologies, Monza, Italy) according to manufacturer’s protocol. cDNAs were then used as a template for quantitative real-time polymerase chain reaction assays. Amplification reaction mix contained 1× iTaq Universal SYBR Green Supermix (Bio-Rad), 400 nM of each primer, and 25 ng of cDNA (RNA equivalent) as template. PCR conditions were 95 °C for 30 s followed by 40 cycles of 95 °C × 5 s and 50 °C × 30 s. Melting curves were generated after amplification using instrument default settings. Data were collected using the CFX Connect real-time PCR detection system (Bio-Rad); each reaction was performed in duplicate. The relative gene expression was calculated using the the 2^−ΔΔ*C*t^ method [39], and *Actb* was used as the housekeeping gene. Primers were designed using Oligo 4.0-s. Sequences of the primers are asfollows:

Cd274 Fw: 5′-AGA GGG GAT GCT TCT CAA TGT G-3′; Cd274 Rv: 5′-CCG TGG ACA CTA CAA TGA GGA A-3′; Itgb3 Fw: 5′-GAA GAA TGC CTG CTT GCC C-3′, Itgb3 Rv: 5′-AGC CCC AGA GAT GGG TAG TC-3′; Irf4 Fw: 5′-GGA GCT GGA GGG ATT ATG CC-3′, Irf4 Rv: 5′-TGC TTG GCT CAA TGG GGA TT-3′; MyD88 Fw: 3′-TTA GGT AAG CAG CAG AAC CAG G-5′; MyD88 Rv: 3′-AGT CTG TCT GTT CTA GTT GCC G-5′; Actb Fw: 5′-TTC TTT GCA GCT CCT TCG TT-3′; Actb Rv: 5′-GCA CAT GCC GGA GCC GTT-3′.

## Figures and Tables

**Table 1:** Summary of the most relevant genes (cited in the manuscript) differentially expressed upon exposure to the two antigen delivery systems.

De-Regulated Status	Differentially Expressed Genes		
	E2	fd-scαDEC-205	Both
*up*	**Extracellular Matrix-receptor interaction:** online Supplementary files **Focal adhesion:** online Supplementary files **Homeostatic chemokines/receptors:** *Ccl25*, *Ccl27*, *Cxcr5*, *Ccr9*, *Ccr10* **Polarization toward DC2:** *Irf4*, *Ccl17*, *Ccr4* **Regulation of actin cytoskeleton:** online Supplementary files **Regulation of cell communication:** online Supplementary files **Regulation of signal transduction:** online Supplementary files	**Chemokine receptors:** *Ccr1*, *Ccr2* and *Ccr5* **Chemotactic chemokines:** *Cxcl1*, *Cxcl2*, *Cxcl3*, *Cxcl10* **DC co-*stimulation*:** *Cd274* **HIN-200 gene family:** see Figure 5 **Inflammatory molecules and chemokines:** *Cxcl10*, *Ccl2*, *Ccl3*, *Ccl4*, *Ccl5*, *Cxcl12*, *Il1a*, *Il1b*, *Il18*, *Ifnar1*, *Ifnar2* **Interferon-induced genes:** *Ifit1*, *Ifit2* **NOD-like receptor** (Nucleotide-binding Oligomerization Domain-like receptors): online Supplementary files **Polarization toward DC1:** *Il12b*, *Il12rb*, *Il6*, *Il6st*, *Ccl3/Mip1a* **OAS gene family** (Oligoadenylate Synthetase): *Oas1a*, *Oas1b*, *Oas1c*, *Oas1g*, *Oas2*, *Oas3*, *Oasl1* **RIG-I-like receptor** (Retinoic acid-Inducible Gene I-like receptor): online Supplementary files **Toll-like receptor:** online Supplementary files	**Chromosome organization:** online Supplementary files **Mediator complex:** *Med12*, *Med13*, *Med13l*, *Med14* **Notch signaling:** *Notch1*, *Notch2* **Regulation of transcription:** online Supplementary files
*down*	**Chemokine receptors:** *Ccr1*, *Ccr2* and *Ccr5* **DC co-*stimulation*:** *Cd274* **Inflammatory chemokines:** *Cxcl10*, *Ccl2*, *Ccl3*, *Ccl4*, *Ccl5*	**Immature DC marker:** *Ccr6*	**Chemokines for basal migration:** *Ccl6*, *Cxcl14*, *Cxcl16* **Immune cells’ survival:** *Il7*, *Pdgfra* **Oxidative phosphorylation:** online Supplementary files **Ribosome:** online Supplementary files

## Supplementary Materials

Supplementary materials can be found at www.mdpi.com/1422-0067/18/3/494/s1.
