# Supplementary material for: Distinct Antigen Delivery Systems Induce Dendritic Cells’ Divergent Transcriptional Response: New Insights from a Comparative and Reproducible Computational Analysis
Source: Int J Mol Sci. 2017 Feb 24;18(3):494. doi: 10.3390/ijms18030494 (PMC5372510; doi:10.3390/ijms18030494)
Supplement: Supplementary file 1 [file ijms-18-00494-s001.pdf]

Supplementary Materials: Distinct Antigen Delivery Systems Induce Dendritic Cells Divergent Transcriptional Response. New Insights from a Comparative and Reproducible Computational Analysis

Valerio Costa, Dario Righelli, Francesco Russo, Piergiuseppe De Berardinis, Claudia Angelini and Luciana D'Apice

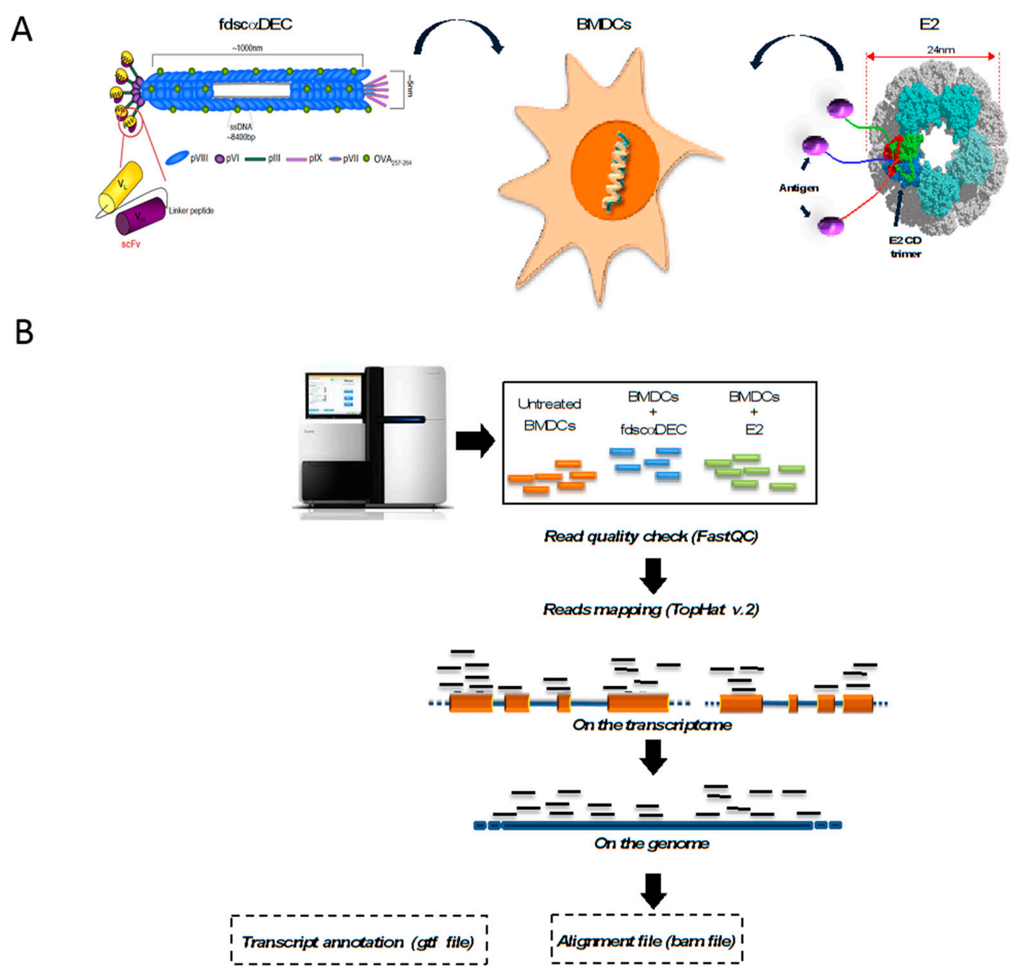

Supplementary Figure *Cartoon of the experimental design.* (**A**) Drawing of BMDC treated with the antigen delivery systems: fd-scαDEC-205 (left) is depicted with the capsid pIII proteins modified to express the single chain fragment of DEC205 antibody to allow the targeting to dendritic cells, the E2 protein (right) is depicted as a self assembled multiprotein complex with the dimension and structure of a viral envelope. (**B**) Schematic workflow of the sequencing strategy and of the mapping procedures. Paired-end reads (in black) have been mapped first against the transcriptome (indicated by orange boxes), then on the genome (blue horizontal bar). Dashed boxes indicate the output files of the mapping procedure. The binary sequence alignment.bam files and the General Transcript File of annotation (.GTF) have been used for the further analyses described in Figure 1.

Supplementary File 1. **http://bioinfo.na.iac.cnr.it/BMDC_analysis/BMDC_analysis_report.html**
